# Supplementary material for: Are carbon emissions trading and green financial instruments synergistic? -Comprehensive quantitative research based on content analysis
Source: PLoS One. 2024 Mar 7;19(3):e0298601. doi: 10.1371/journal.pone.0298601 (PMC10919601; doi:10.1371/journal.pone.0298601)
Supplement: S1 Data — (ZIP) [file pone.0298601.s001.zip › Data/地级市Z_F工作报告.zip!/╔┬╬≈/╧╠╤⌠╩╨/└┤╫╘╣1⁄2╓┌║┼í╛┬φ┐╦╩2╛▌═°í┐_2016.docx]

各位代表：

现在，我代表市人民政府向大会作工作报告，请予审议，并请市政协委员及列席人员提出意见。

一、2015年和“十二五”工作回顾

2015年是我们团结拼搏、克难奋进的一年。面对复杂严峻的经济形势，我们认真贯彻“三稳两优”重大部署，围绕追赶超越，突出“五抓”举措，实施对标管理，推动经济社会平稳健康发展。全市生产总值完成2155.9亿元、增长8.7%；财政总收入和地方财政收入分别完成218.7亿元、85.4亿元，增长11.9%、16.1%。城乡居民人均可支配收入达到29425元和9690元，分别增长8.4%、9.5%。我们主要做了以下工作：

一是积极应对下行压力，经济保持平稳运行。加强分析研判，抓住重点领域和关键环节，采取有效措施，着力破解经济运行中的突出问题。坚持稳产促销保工业，扎实推进“1113”工程，启动工业翻番计划，强化“八抓八保”措施，全力支持企业稳定产能、拓展市场，全市规模以上工业增加值完成974.3亿元、增长9.2%。立足提质增效稳农业，以农业十大工程为抓手，发挥示范园区带动作用，调整产业结构，支持龙头企业发展，认真解决果品、鲜奶等销售难题，粮食生产实现“十二连丰”，新发展矮化苹果7.6万亩、设施蔬菜2.3万亩，肉蛋奶稳定增长。着眼壮大三产扩消费，实施电子商务发展规划，武功和泾阳入列全国农产品电子商务示范县，电子商务交易规模突破100亿元、增长67%；乡村旅游接待游客1550万人次，全市旅游综合收入达到271.6亿元，社会消费品零售总额完成601.6亿元、增长13.8%。

二是坚持扩投资调结构，有效集聚发展势能。全社会固定资产投资完成3063.2亿元、增长22.9%，高出全省15.1个百分点；民间投资占比达到60%，经济活力进一步增强。集中力量加快项目建设，190个市级重点项目共完成投资925.2亿元，占年计划的121.2%。强化服务支持，西咸新区50个项目完成投资173.7亿元。积极推进战略招商、精准招商，主动加强与央企、省企对接，全年共引进合同项目252个、合同资金1965.9亿元；实际完成投资837.3亿元。陕韩中小企业园开局良好，泰迪熊博物馆落户大西安文体功能区。总投资280亿元的CEC·咸阳8.6代液晶面板生产线项目开工，电子信息产业转型升级迈出关键一步。

三是坚持规划引领、拉大框架，全力建设“大西安”核心区。抢抓关中城市群建设机遇，扎实推进“一市五县”建设，16个重点示范镇、文化旅游名镇完成投资22.9亿元。着眼优化城市形态、完善城市功能，加快大西安文体功能区建设，市民文化中心主体建成，省十六运主场馆咸阳奥体中心规划设计完成，北塬一路、平福大道即将通车。扎实推进中心城市“6+3”工程，启动三年改造提升计划，年度投资79亿元的148个重点城建项目顺利实施，文兴西路、丝路公园等项目先后建成，公交一卡通正式启用，一批地下管廊和停车场启动建设，点亮工程成效明显，夜色中的咸阳更加迷人。国家卫生城市复审顺利通过，全国文明城市创建工作扎实开展，“崇德包容、尚法创新”的咸阳精神深入人心。

四是突出系统治理，生态环境质量持续好转。坚持铁腕整治、建管并举，全力打好治污降霾攻坚战，完成治污降霾重点项目681个，全年市区空气质量优良天数达到258天、较上年增加41天。渭河水污染防治三年行动全省考核第一，旱腰带采石矿山治理全面启动，马栏河、冶峪河、清峪河3个国家级湿地公园试点通过验收，创建全国绿化模范城市顺利通过国家绿化委核查。狠抓节能减排，加大重点企业提标改造力度，四大污染物排放量得到有效控制。

五是扎实办好惠民便民实事，人民群众有了更多的获得感。30项惠民便民实事全面完成，民生支出达到251.7亿元，占财政支出的83.8%。城镇新增就业6.2万人，城镇登记失业率控制在3.21 %以内。扩大大病保险、困难救助覆盖面，调整提高8大类21项社会保险待遇标准。精准扶贫第一批146个整村推进项目全面完成，旱腰带区域特色城镇化移民搬迁10个项目有序推进。市第一人民医院、市中心医院扩建步伐加快，全年医保报销31.7亿元。全市新建和改扩建中小学、幼儿园405所，兴平、长武、秦都3个县市区通过省级双高双普评估验收，全省职业教育工作推进现场会在我市召开。改造、新建永寿、三原、礼泉等3个县级体育场馆。积极创新社会治理，平安创建工作深入推进。深刻汲取5.15事故教训，加强重点领域安全隐患排查整治，社会大局保持稳定。

六是全面深化改革，持续增强经济发展活力。完成市县机构改革任务，推行权力责任清单，深化行政审批事项改革及商事制度改革，加快网上政务中心建设，群众办事更加方便。公车改革市县同步推进，全市“三公”经费下降16%。深化金融改革，通过设立企业担保贷款风险补偿金，撬动金融资本13.6亿元，中小企业融资难问题得到缓解。镇村综合改革、社会组织管理体制改革扎实推进，农村集体建设用地和宅基地发证率达到85％和83％。加快创新型城市建设，202所李魁武同志当选中国工程院院士，科技型企业科隆橡胶在新三板成功上市，技术合同交易总额30.7亿元、增长19%。

一年来，双拥、老龄、残疾人、红十字、妇女儿童等事业取得了新进步，外事、侨务、对台、审计、统计、民族、宗教、地震、气象、人防、档案、地方志等工作实现了新发展。

各位代表！2015年各项工作的顺利推进，标志着我市“十二五”规划实现圆满收官。回顾过去的五年，我们克服了诸多困难，战胜了严峻挑战，办成了许多打基础的实事、利长远的大事、补短板的难事，咸阳改革开放和现代化建设取得新的重大成就。

——五年来，我们扭住发展第一要务不动摇，加快追赶超越，综合实力大幅提升。生产总值年均增长12.3%，比2010年净增1057.2亿元，经济总量由西部城市的第15位前进到13位。规模以上工业增加值、全社会固定资产投资、社会消费品零售总额、地方财政收入、各项存款余额年均增长17.5%、24%、15.2% 、17.3%、14.6%，分别是2010年的2.3倍、2.9倍、2倍、2.2倍和2倍。秦都进入全省五强区，渭城名列第七。彬县、兴平进入全省十强县，三原、泾阳、礼泉、乾县进入全省前20名。

——五年来，我们坚持矢志不移调结构，一手抓招商引资扩增量，一手抓科技创新提质量，积极探索咸阳又好又快发展新路子。全市三次产业结构调整到15.3:57.5:27.2，科技对经济的贡献率提高到54%。农业总产值稳居全省第一，农业机械化水平大幅提升，传统农业加速向优质高效、生态观光农业转变。工业主导地位持续增强，华晨汽车、正泰电气、修正药业、双汇等项目助推装备制造、食品、医药产业加快发展，高新区升级国家级开发区，彬长旬高端能化基地建设为加快煤炭转化奠定了坚实基础，纺织工业通过退城入园焕发生机。实施服务业三年行动计划，现代物流、电子商务等生产性服务业迅猛发展，乡村旅游竞相发展、各具特色，成为咸阳一张靓丽名片。

——五年来，我们以共建“大西安”为统揽，强化基础设施建设和生态环境保护，城乡面貌发生显著变化。加强与西安、西咸新区的基础对接和产业联动，沣西新城、空港新城、秦汉新城、泾河新城创新发展，为我们稳增长、调结构发挥了重要支撑作用。咸旬高速建成实现了县县通高速，西咸北环线、旬铜高速通车，标志着咸阳绕城高速全面建成、境内高速连接成网，地铁1号线二期、西安北客站至机场轨道交通启动实施，西宝高铁、西平铁路客货运全线开通，西安（咸阳）国际机场旅客吞吐量突破3000万人次，现代立体交通体系基本形成。持续推进棚户区改造， 全市10.5万群众告别老旧危房、喜迁新居。大力实施畅通工程，打通城市断头路，新建道路118.3公里，改造道路30.3公里，中心城市建成区面积扩大到90.6平方公里，城市绿化率提高到33.6%，成功创建国家卫生城市、国家园林城市、省级环保模范城市。渭河综合整治工程全面完工，咸阳湖二期启动实施，渭河成为安澜河、生态河、景观河的目标正在变成现实。城乡一体化步伐加快，新农村建设扎实推进。东庄水库、亭口水库等重大水利设施开工建设，发展支撑能力显著增强。

——五年来，我们坚持以改革增活力，不断创新体制机制，“五型”政府建设扎实推进。紧紧围绕建设学习型、法治型、服务型、务实型、廉洁型政府，加快转变职能、简政放权，累计取消行政审批事项81项、承接97项、下放38项，市场准入门槛降低，营商环境明显改善。扎实开展大众创业、万众创新活动，市场主体五年净增9.6万户，非公经济占比达到52.1%。全面落实从严治党各项部署，认真开展群众路线教育实践活动和“三严三实”专题教育，政府效能进一步提升，我市干部作风满意度在全省测评中名列前茅。实行镇村群众办事干部代办制度，提高农村两委会干部待遇。坚持依法行政，自觉接受人大依法监督和政协民主监督，共办理人大代表建议944件、政协委员提案3262件。

——五年来，我们始终把改善民生作为头等大事，更加注重兜底线、补短板，广大群众生活水平不断提升。持续加大财政对民生投入，坚持两个80%不动摇，每年实施一批惠民便民实事，事关群众切身利益的热点难点问题得到逐步解决。制定出台教育、卫生、文化、体育等社会事业发展规划，基本公共服务保障能力明显增强。覆盖城乡的社会保障体系基本建立，基本养老和基本医疗保险覆盖率达到96%。累计搬迁贫困群众15.6万人，稳定脱贫48.5万人，建设保障性住房20.1万套，解决了农村266.2万人的饮水不安全问题。城乡居民收入分别比2010年增加15273元、5473元。

各位代表，我市“十二五”时期改革发展成就来之不易，这是省委、省政府和市委正确领导的结果，是市人大、市政协依法监督、民主监督的结果，是全市广大干部群众攻坚克难、开拓奋进的结果。在此，我代表市人民政府，向全市人民、驻咸部队、武警官兵和公安民警，向大力支持政府工作的各位代表、各位委员和社会各界人士，表示崇高的敬意和衷心的感谢！

“十二五”发展成就令人鼓舞，但前进道路上还面临不少困难和问题。主要是：经济下行压力仍然较大，结构性矛盾比较突出，创新驱动发展能力不强，发展方式比较粗放，一产不优、二产不强、三产不足的问题尚未得到根本解决；城乡发展不协调，城乡居民收入差距比较大，公共服务不均衡，仍有近33万人口还处于贫困线以下；社会治理能力亟待提升，生态环境、安全生产与群众期盼还有一定差距；政府职能转变还不到位，依法行政的能力有待提高，新常态下一些干部不敢担当、不会作为。对这些问题，我们将采取有效举措加以解决。

二、“十三五”时期的总体思路和目标任务

按照党的十八届五中全会、中省“十三五”规划《建议》和市委六届十二次全会精神，市政府编制了“十三五”规划纲要（草案），提出了我市“十三五”时期经济社会发展的指导思想、总体目标和主要任务，本次大会审议批准后，我们将认真组织实施。

各位代表，“十三五”时期，是咸阳发挥优势实现追赶超越的关键期，是破解难题加快转型升级的攻坚期，是补齐短板全面够格建成小康社会的决胜期。冷静分析宏观形势，我国经济进入速度换挡、结构调整、动力转换的新常态，生产能力、生产方式、生产关系深刻调整，经济发展的不确定性、不稳定性较大，但长期向好的基本面没有改变。随着“一带一路”战略的深入实施，省市共建大西安和关中城市群建设的扎实推进，加之多年来集聚的产业基础和发展后劲，我们完全有条件、有优势实现追赶超越，开创经济社会发展新局面。

“十三五”的奋斗目标是：建设富强人文健康新咸阳。

——富强咸阳。经济保持中高速增长，产业结构更趋合理，创新引领发展格局基本形成。在质量效益明显提高的基础上实现生产总值年均增长9%左右，到2020年人均GDP超过1万美元，规模以上工业增加值年均增长12%左右，社会消费品零售总额年均增长12%左右，三次产业结构进一步优化。非公经济增加值占GDP比重达到58%，战略性新兴产业和服务业增加值占GDP比重进一步上升，税收收入占财政收入的比例达到70%以上，居民人均可支配收入年均增长10%。

——人文咸阳。秦风秦韵秦文化进一步彰显，“崇德包容、尚法创新”的咸阳精神成为广大市民的自觉行动，“大秦故都、德善咸阳”的城市品牌更加靓丽，咸阳软实力明显提升。全力创建全国文明城市，公民思想道德素质、科学文化素质、法治素质、文明素质和社会文明程度明显提高。到2020年，公共文化服务体系基本建成，文化产业增加值突破200亿元，比重达到6%。旅游产业规模进一步壮大，旅游业总收入近500亿元，年均增长13％。

——健康咸阳。经济、政治、文化、社会、生态等各个领域协调发展。城乡一体化发展格局基本形成，对外开放水平和经济可持续发展能力明显增强。医药卫生体制改革取得重大突破，社会保障体系更加健全，基本公共服务实现均等化。生态环境质量全面改善，美丽咸阳建设深入实施，全市森林覆盖率达到39%，湿地保护面积达到15.4万亩，城市污水处理率达到95%以上。社会治理能力进一步提升，依法治市全面推进，群众安全感和幸福感显著增强。

实现富强人文健康新咸阳的目标，必须牢固树立创新、协调、绿色、开放、共享发展理念，把改革创新作为政府工作第一追求，以推进供给侧结构性改革为突破口，坚决跳出传统发展模式，加快实现由投资拉动向创新驱动转变，由量的扩张向量质并重转变，推动发展实现中高速增长，迈向中高端水平；坚决转换发展动能，突出结构调整助推产业转型升级，突出科技创新培育发展新的动能，突出兜底补短形成共建共享格局，不断加快追赶超越步伐；坚决破除惯性工作方式，认识新常态、适应新常态、引领新常态，以市场手段配置资源，以法治思维服务发展，确保同步够格全面建成小康社会，为“三个陕西”建设贡献咸阳力量。重点做好五个方面工作：

坚持以创新为引领，增强产业发展新动力。优化存量与扩张增量齐抓并举，一二三产协调推进，供给消费同步发力，形成以创新为引领的现代产业体系。实施工业翻番计划，打造能源化工、装备制造、食品三个千亿产业，电子信息、医药、纺织、建材、战略性新兴产业五个百亿产业。推动生产性服务业向专业化和价值链高端延伸、生活性服务业向时尚精细和高品质转变，着力培育现代物流、电子商务、健康养老、文化旅游等新的经济增长点。加快推进农业现代化，着力构建现代农业生产体系、经营体系，促进农业与二产、三产融合发展，形成优质高效、生态观光、绿色安全的现代农业发展格局。以建设国家创新型城市和知识产权示范城市为抓手，强化企业主体地位，实施重大科技项目，促进创新链与产业链、资本链、服务链有机融合，加快构建富有咸阳特色的科技创新体系。

坚持以协调为关键，构筑均衡发展新格局。加快规划对接和基础设施建设，与西安、西咸新区携手共建现代化“大西安”新中心。统筹区域发展，加快县域经济争先进位，彬县、兴平确保10强位次并实现进位，三原、泾阳力争进入全省10强。扎实推进以人为核心的新型城镇化，努力打造“一主一副双核心和两环两轴多辐射”城镇体系，狠抓新型农村社区、传统村落保护和美丽乡村建设，常住人口城镇化率达到60%。新改建国道481公里、县乡公路1492公里，全面建成亭口水库、红岩河水库、柏岭寺水库、羊毛湾引水以及张家山泉群开发供水工程。

坚持以绿色为方向，建设生态文明新家园。严格划定并执行各类生态红线，统筹安排生产、生活、生态用地，控制建设用地总规模，认真落实自然资源、环境保护督察和终身追责制度，全面促进资源节约利用，形成人与自然和谐发展新格局。坚决打好大气、土壤、水污染防治三大战役，实施“治污降霾·保卫蓝天”五年行动计划，推进电能替代，加大渭河、泾河、沣河生态治理力度，系统谋划建设全市水系和湿地，加快旱腰带区域生态修复和北部绿色矿山建设，深入实施“北部森林化、中部果林化、南部园林化”生态工程。生活垃圾无害化处理率达到 95%以上，主要污染物排放明显下降，单位生产总值能耗累计下降16%。

坚持以开放为动力，打造改革开放新高地。统筹对内开放和对外开放，积极融入“一带一路”建设，转变招商引资方式，实施项目带动战略，把咸阳建成国际航空枢纽和临空产业示范基地、国家现代农业和食品工业基地、西部重要的现代工业基地、西部区域性商贸物流基地、大西安科技成果应用转化基地和秦汉唐文化交流展示基地。全面深化各项改革，下好简政放权先手棋，深入推进重点领域和关键环节改革，形成系统完备、科学规范、运行有效的制度体系，推动经济社会持续健康发展。

坚持以共享为根本，汇聚全面小康新合力。坚持以人为本，完善制度安排，强化政策兜底，让人民群众更多分享改革发展成果。大力推进大众创业万众创新，深入实施居民收入倍增计划。普及学前3年教育，全面实施13年免费教育，推进中等职业教育免费。社会保障实现全覆盖，社会救助体系更加完善，形成满足多层次需求的住房供应体系。坚决打赢脱贫攻坚战，两年脱贫、三年巩固，全面实现建档立卡贫困人口脱贫、四个贫困县摘帽。

各位代表，机遇承载使命，挑战考验担当。只要我们尊重人民、依靠人民，以燕子垒窝的恒劲、蚂蚁啃骨的韧劲、老牛爬坡的拼劲，坚定信心、埋头苦干，就一定能够实现富强人文健康新咸阳的奋斗目标，就一定能够夺取全面建成小康社会的伟大胜利！

三、2016年经济工作重点任务

今年，我们要积极适应经济发展新常态，统筹推进稳增长、调结构、促改革、惠民生、防风险各项工作，着力加强供给侧结构性改革，切实抓好去产能、去库存、去杠杆、降成本、补短板五大任务，确保“十三五”开好局、起好步。

预期目标是：生产总值增长9%左右，全社会固定资产投资增长15%左右，规模工业增加值增长12%左右，社会消费品零售总额增长11%左右，地方一般公共预算收入同口径增长10%左右，城乡居民人均可支配收入分别增长9%、10%左右，城镇登记失业率控制在3.9%以内。

（一）突出供给侧结构性改革，增强经济持续发展动力

坚持去产能、调结构并重。核心是通过调结构消化无效产能、培育崭新动能，加快产业转型升级步伐。转变农业发展方式，推进农业提质增效，实施品牌化战略，提升农产品市场竞争力，加快传统农业向优质高效、生态观光农业转变。以彬长旬高端能源化工基地建设为抓手，主动加强与神华、中石化、延长、陕煤等大型企业对接，引进重大转化项目，延伸产业链条，提高煤炭产业附加值；严格执行产业规划，严控建材产能；加快国企改革步伐，坚持市场化导向引进战略投资，争取中央专项奖补资金，坚决淘汰“僵尸企业”，对国有资产进行审计，不断优化国有资本配置。立足大西安核心区，统筹规划、错位发展，着力壮大物流、电子商务等生产性服务业，加强孵化基地和产业园区建设，打造重要物流节点城市。

扎实开展“三个专项行动”。坚持分类施策改善消费品供给，以食品、医药产业为重点，进一步完善支持政策，推动企业开发适销对路新产品，提升终端产品供给的层次和水平。实施质量强市战略，鼓励企业争创品牌、拓展市场。加快纺织企业升级改造，大力发展高附加值纺织产品，积极推进与中亚国家的产能合作。多措并举促进降本增效，积极争取省上实施直供电和低电价区改革试点，加快实施涉企收费目录清单管理，清理规范中介服务，从交易、人工、税费、财务成本等方面给企业松绑减负。超前谋划制造业升级，抢抓“中国制造2025”行动计划重大机遇，培育发展新能源汽车、智能制造等高端装备制造产业。加快CEC·咸阳8.6代线项目建设，促进电子信息产业资源整合、转型升级，加快形成产业集群。

积极推进要素资源改革。加大闲置土地处置力度，盘活存量土地，优先确保重大基础设施、战略性新兴产业和民生项目用地。设立电子信息、装备制造等产业发展基金，通过整合财政资金、推行股权投资等方式，撬动社会资本投向产业。深化金融改革，支持发展普惠金融、绿色金融，加快地方银行组建步伐，健全金融组织体系，鼓励企业直接融资，提升金融服务实体经济能力。全面实行居民用电、用水、用气阶梯价格，加快形成供水、供气、供热等方面的价格市场化决定机制。

着力做好“放、管、服”工作。进一步推进简政放权，全面公布市级权力清单和责任清单，制定市场准入负面清单。巩固“多证合一、一照一码”，积极探索“证照分离”，继续减少和规范行政许可事项。推进工商、质监、食品药品监督执法重心下移，切实加强对市场秩序、安全生产、食品药品安全、社会诚信领域的监管。建立健全投资环境评价机制和社会监督机制，严肃整治“三乱”以及强揽工程行为，坚决斩断伸向企业的黑手，全面提升执法队伍素质，努力营造良好的营商环境。

提高科技创新能力。围绕创建国家创新型城市，发挥科研院所和军工企业较多的优势，促进“两化”融合、军民融合，加速科技成果转化应用，培育形成新的经济增长点。加大财政资金对科技创新的支持力度，推进科技与金融结合，建立科技成果供需对接机制，实施科技创新人才培育工程。向国内一流开发区看齐，推动高新区、大西安文体功能区建设创新服务孵化平台，支持三原高新技术产业开发区加快发展。

（二）突出稳中有进、稳中有为，促进经济在合理区间运行

力保工业稳增长。坚持工业主导不动摇，扎实推进“工业翻番”计划，继续实施“1113”工程，推动工业经济持续健康发展。以七大支柱产业为重点，强化职能部门包抓责任，支持优势企业上规模、上水平。加快神华国能彬长低热值煤示范项目前期工作，确保大唐彬长电厂二期等项目年内开工，扎实推进100个技改扩能项目、100个工业重点项目建设，尽快形成新的产能。实施个转企、小升规培育计划，年内新增规模以上工业企业100户，培育40户科技小巨人企业，增强工业经济实力。

夯实农业稳增长。综合发挥市场带动、园区示范、政策引导、项目支持作用，打造粮果畜菜特色产业板块。认真落实粮食安全战略，稳定粮食总产，提高种粮效益。按照稳面积、抓改造、提品质、创品牌的思路，大力推广矮化苹果种植，持续增强苹果产业竞争力。大力发展农村电子商务，打响“马栏红”苹果、“泾阳蔬菜”等特色农产品品牌，扩大咸阳茯茶等优势产品市场份额。深度挖掘农业多种功能，着力推进乡村旅游与农业产业融合发展，引导农业园区向景区升级，促进农产品向旅游商品转化。加快咸阳·杨凌农业产业一体化发展，建设一批农业高新科技试验示范基地。进一步深化农村产权等各项改革，促进生产要素投向农业农村，支持龙头企业、种养大户、专业合作社、家庭农场发展。完善水利设施，加强土地治理和生态修复，扎实推进全国农业机械化示范市建设，着力改善农业生产条件。

壮大三产稳增长。把旅游文化产业作为支柱产业来培育，探索建立旅游文化产业投融资平台，加强精品景区和线路开发，促进乡村旅游提档升级和特色化发展，有效提升文化旅游产业影响力和竞争力。发挥中医资源富集优势，引导民营资本和公立医院发展养老产业，推进健康产业与养老、文化旅游、房地产等产业深度融合。加强市场监管，抓好特色商贸街区及村镇建设，打造一批便民消费示范社区，促进人气集聚和业态繁荣。着力化解房地产库存，允许农业和转移人口等非户籍人口在就业地落户，支持自住性、改善性住房消费。

做强县域稳增长。支持彬县建设市域副中心城市，加快撤县设市步伐，推进彬长旬地区协同发展、板块联动，促进武功、兴平、秦都、渭城等县市区联手打造沿渭装备制造产业带，鼓励乾县、礼泉、泾阳、三原建设大西安产业配套基地，扶持淳化、永寿和旬邑建设大西安避暑后花园和天然氧吧。支持县域工业园区创新发展，力争更多的县域工业集中区升级为省级重点园区，打造支撑县域发展的动力极。

（三）突出招大引强、项目带动，着力提升对外开放水平

扭住招商引资不放松。紧紧抓住新丝绸之路经济带建设机遇，突出创新招商、精准招商、叩门招商，紧盯世界500强、国内民营100强，严格执行“五个不要”清单，一手抓资本技术密集型龙头项目，一手抓劳动密集型产业项目，提高招商引资的质量和效益。围绕CEC8.6代线上下游产业，加大项目招引力度，加快形成完整产业链。积极借助丝博会暨西洽会、厦洽会、陕粤港澳经济合作活动周等平台，抓好央企进陕、陕企进咸项目对接落地，力争全年招商引资到位资金达到890亿元。

狠抓重点项目建设。今年市级重点建设项目计划安排170个，年度投资656.8亿元。其中续建项目81个，年度投资308.1亿元；新开工项目89个，年度投资348.7亿元。严格落实“五个一”项目工作机制，认真解决审批、征地拆迁等方面问题，推进咸阳汽车商贸物流园、修正药业西北生产基地、国电咸阳热电联产等项目加快建设。主动对接中省各项规划及重大政策，及时策划包装项目，积极争取上级资金支持。

加强平台建设。积极服务西咸新区加快发展，支持沣西新城建设国家大数据产业示范基地、空港新城建设国家航空城试验区、秦汉新城建设省文化产业示范基地、泾河新城建设国家现代农业示范区，全力做好新区项目建设服务保障。抓好高新区、新兴纺织工业园、彬长旬高端能化基地基础设施建设，支持大西安文体功能区申报省级经济技术开发区，提升园区承载能力，打造对外交流合作平台。突出纺织、建材、食品及现代农业，引导企业加强与中亚地区的产能合作，促进“丝绸之路文化产业园”项目尽快开工。进一步优化外贸进出口结构，力争全年外贸进出口总额实现28.8亿元，提升经济外向度。

（四）突出统筹城乡、生态宜居，不断优化城乡发展空间格局

提升中心城市品质。对接关中城市群核心区规划，争取市县两级全域规划、“多规合一”试点，修编完善中心城市总体规划，注重城市设计，完善城市功能。切实抓好西安北客站至咸阳机场轻轨、地铁一号线二期工程、咸阳高铁客运枢纽站建设工作。加快大西安文体功能区建设，推进奥体中心、双照水库开工建设，不断增强聚集承载能力。扎实推进中心城市“6+3”工程，精心组织实施主城区三年改造提升计划，争取海绵城市、地下综合管廊城市试点，加快棚户区改造步伐。积极筹划“三纵一横”城市快速道路，实施文兴东路、咸北大道等路桥工程，推进城市亮化由咸阳湖北岸向南岸和人民路主要商业区扩展，抓好城市绿化景观建设，让城市更加时尚、更加宜居。继续加强智慧咸阳建设，实施大数据城市信息融合示范工程，发展民生服务智慧应用。巩固提升创卫、创园成果，扎实开展全国文明城市创建工作。加快推进城市执法体制改革，坚持权责一致，下移执法重心，充实一线力量，提升城市精细化管理水平。

加快城乡一体发展。以关中城市群“一市五县”为重点，突出地域特色，优化功能分区，加强市政设施以及公共服务、生态环保设施建设，提升县城辐射带动能力。继续加强16个省市重点镇及文化旅游名镇建设，积极开展扩权强镇试点，促进园区、产业、人口聚集，着力培育引进劳动密集型产业，打造一批工业强镇、商贸名镇。加快农业转移人口市民化，积极做好教育、就业、医疗、养老、住房保障等工作，使新市民进得来、留得住、能融入。加强城乡基础设施建设，实施国道108三原至礼泉一级公路、国道211淳化、312永寿、344兴平等过境道路升等改造，促进城乡联动发展。

持续优化生态环境。坚持铁腕整治、久久为功，在“治污降霾”上狠下功夫，全力推进减煤、控车、抑尘、治源、禁燃、增绿等工作，确保空气环境质量持续改善。扎实推进新一轮渭河三年行动计划，进一步加大湿地保护和生态恢复力度，加快咸阳湖二期、泔河乾礼段生态湿地、泾河生态综合整治等项目建设，切实做好滩涂整治绿化工作。深入实施“旱腰带”地区生态修复治理工程，积极发展光伏发电、畜牧养殖、景观旅游等产业，把旱腰带打造成大西安的“绿腰带”、促进群众增收的“金腰带”。开展农村环境综合整治示范工作，巩固提升创绿成果，全年营造林30万亩，绿化道路300公里。

（五）突出脱贫攻坚、兜底补短，着力保障和改善民生

全面打响脱贫攻坚战。以六盘山片区、4个国家扶贫重点县、革命老区县以及旱腰带地区为主战场，抓住精准识别、规划引领、以人为本三个关键，突出发展生产“改穷业”、易地搬迁“挪穷窝”、教育培训“拔穷根”、生态保护“换穷貌”、兜底保障“脱穷境”五个重点，整合资源，因人施策，精准扶贫、精准脱贫，确保20.7万人稳定脱贫。统筹做好城市扶贫工作，切实提高城区困难群众生活水平，实现脱贫攻坚无盲区，城乡群众全覆盖。

完善公共服务。扎实推进“双高双普”工作，加快构建现代职业教育体系，全面完成第二期学前教育三年行动计划。深化医药卫生体制改革，抓好公立医院综合改革，健全分级诊疗制度，发展智慧医疗，提升基层医务人员专业技术水平。全面实施一对夫妇可生育两个孩子政策，切实落实失独家庭补助救助政策。办好第十一届中国文化艺术节，健全现代公共文化服务体系。启动奥体中心建设，加快渭河沿岸全民健身长廊建设，推进各县市建设标准体育场，实行全市中小学操场对外开放，深入开展创建全民健身示范市和全民健身活动。

加强社会保障。做好就业创业工作，完善扶持政策，鼓励自主创业，农村劳动力转移就业规模保持在100万人次以上，城镇新增就业4.9万人。完善社会保障机制，做好灵活就业人员及农民工参保工作，推进城乡居民基本医疗保险制度整合，提高经办服务水平。健全社会救助制度，完善城乡特困人员供养制度，实现动态管理下的应保尽保。制定养老服务体系规划，建成社区日间照料中心和农村互助幸福院200个。精心实施保障性安居工程，确保项目竣工前六个月编制完成配租配售方案、验收后三个月分配入住。落实农村留守儿童、妇女、老年人关爱政策，健全残疾人权益保障制度，妥善安排好困难群众生产生活。

积极创新社会治理。全面启动“七五”普法，加快县市区法律服务中心建设，加强特殊人群服务管理。进一步深化信访制度改革，着力打造阳光信访、责任信访、法治信访，切实维护群众合法权益。加强双拥工作，巩固和发展军政军民团结。全面落实党政同责、一岗双责、失职追责，继续深化安全生产“三化”建设，严防各类事故的发生。推行网格化管理，提升社区服务水平。加强社会治安综合治理，严厉打击各类违法犯罪活动，确保社会大局和谐稳定。

各位代表，为了让群众生活得顺心舒心，我们坚持每年办好一批惠民便民实事。今年我们将投资100亿元，继续办好30件惠民便民实事。围绕提升群众健康保障水平，开工建设市中心医院医技综合楼、两所县级中医院医技综合楼、10个乡镇卫生院医技楼以及220个村卫生室项目，加快重大疾病防治设施建设，让群众享受更加优质的医疗服务。围绕提高教育教学质量，实施基础教育助学、职业教育助学、双高双普以及特殊教育惠民工程，新建彩虹第二中学、英才学校、渭城中学、渭城职教中心，迁建市特殊教育学校，扩大优质教育资源，缩小区域、城乡和校际差距，促进义务教育均衡发展。围绕方便群众出行，支持公交优先发展，投放100辆公交车、1000辆公用自行车，加快推进西咸公交一卡通，出台城市停车设施和电动汽车充电桩建设规划，实施一批城市公共停车设施工程。

群众关心的事就是政府的头等大事，群众对幸福生活的企盼就是政府的不懈追求，我们将始终坚持群众利益至上，及时回应群众关切，着力解决群众困难，努力让孩子们快乐成长、让青年人乐业创业、让老年人颐养天年，让咸阳的父老乡亲们生活得更加美好、更加幸福！

四、切实加强政府自身建设

新常态下实现追赶超越，需要倍加努力、不懈奋斗。我们要巩固党的群众路线教育实践活动成果，自觉践行“三严三实”，提升依法行政能力，持续改进工作作风，加快推进政府治理体系和治理能力现代化。

坚持依法行政，健全政府责任体系。牢固树立法治理念，严格按照法定权限和程序行使权力、履行职责，完善重大行政决策程序，健全公众参与、风险评估、合法性审查等制度。推进执法重心向基层下沉，切实做到严格规范执法。加大财政预算、重大项目、公共资源等重点领域信息公开力度，做到决策公开、服务公开、结果公开。自觉接受人大及其常委会的法律监督、工作监督和政协的民主监督，主动接受公众监督和媒体监督。

注重改革创新，提升政府服务效能。坚持解放思想、与时俱进，积极探索新常态下做好经济工作的方法和经验，着力推进观念创新、体制创新、管理创新和服务创新。加快转变职能、简政放权，把该放的坚决放开，把该管的坚决管好。推广“互联网+政务服务”，推行网上行政审批、综合执法、公共资源交易等应用系统，坚持县乡村三级群众办事干部代办制度，让群众和企业少走路，让信息和服务多跑路，最大限度地服务企业、方便群众。

着力转变作风，打造人民满意的公务员队伍。健全政府系统干部学习制度，加强公务员培训和挂职锻炼，提高战略思维和实践创新能力。加大治庸问责力度，坚决查处“乱作为”，大力治理“不作为”，着力解决“不会为”，确保令行禁止、执行有力。以目标责任考核为抓手，深化对标管理，建立合理容错机制，支持干部敢于担当、争先创优。大力弘扬“马上就办、办就办好”的优良作风，用心谋事、务实干事、努力成事，以倒计时的状态抓好工作落实。

强化监督制约，推进廉洁政府建设。严格落实“一岗双责”主体责任，进一步加强党风廉政建设，健全审计监督、廉政监察和效能监察机制，严肃查处各种违纪违法行为，着力解决群众身边的腐败问题。深入贯彻厉行节约、反对浪费各项规定，强化“三公”经费管理，不断降低行政成本。加强廉政教育，培养高尚的职业道德和精神追求，树立为民、务实、清廉的良好形象。

各位代表，2016年是“十三五”开局之年，新的征程催人奋进，新的任务艰巨光荣。让我们在市委的坚强领导下，牢记广大群众的期盼和重托，振奋精神，真抓实干，为建设富强人文健康新咸阳而努力奋斗！
